# Clinical and Pathological Characteristics of Bladder Cancer in Patients Aged 18–45 Undergoing Transurethral Resection of Bladder Tumor

**DOI:** 10.3390/biomedicines12112449

**Published:** 2024-10-25

**Authors:** Alexei Croitor, Vlad Dema, Silviu Latcu, Razvan Bardan, Dorin Novacescu, Vlad Barbos, Alis Dema, Alin Cumpanas

**Affiliations:** 1 Department XV, Discipline of Urology, “Victor Babes” University of Medicine and Pharmacy, 300041 Timisoara, Romania; alexei.croitor@umft.ro (A.C.); silviu.latcu@umft.ro (S.L.); razvan.bardan@umft.ro (R.B.); cumpanas.alin@umft.ro (A.C.); 2 Department II of Microscopic Morphology, Victor Babes University of Medicine and Pharmacy Timisoara, 300041 Timisoara, Romania; novacescu.dorin@umft.ro; 3 Angiogenesis Research Center, Victor Babes University of Medicine and Pharmacy Timisoara, 300041 Timisoara, Romania; 4 Department of Urology, Emergency County Hospital Oradea, 410169 Oradea, Romania; vlad.barbos@umft.ro; 5 Department of Microscopic Morphology-Morphopatology, ANAPATMOL Research Center, “Victor Babes” University of Medicine and Pharmacy, 300041 Timisoara, Romania; dema.alis@umft.ro

**Keywords:** TURBT, bladder cancer, oncology, urology

## Abstract

Background and Objectives: Bladder cancer in patients under 45 is poorly characterized and rarely described, with variabilities in clinical outcomes and tumor properties. Our study aimed to elucidate the clinical and pathological features and outcomes of bladder cancer in this younger demographic to better inform management strategies. Materials and Methods: We conducted a retrospective analysis at the Urology Department of “Pius Brînzeu” County Emergency Clinical Hospital in Timișoara, Romania, on 60 patients aged 18–45 who underwent transurethral resection of bladder tumor (TURBT) during a 9-year period. Results: The cohort had a mean age of 38.5 ± 5.6 years with a male predominance (70%). Most tumors were non-muscle-invasive (NMIBC; 80%), with 16.7% being papillary urothelial neoplasms of low malignant potential (PUNLMP), 50% stage pTa, and 30% stage pT1. High-grade tumors were present in 43.3% of the patients. Recurrence occurred in 40% of the patients, while progression was observed in 16.7%. The 3-year overall survival rate was 93.3%, and the progression-free survival rate was 83.3%. Patients with high-grade tumors had a significantly higher recurrence rate (61.5% vs. 23.5%, *p* = 0.003) and lower survival rates compared to those with low-grade tumors. Conclusions: Young patients predominantly present with low-to-intermediate-stage tumors, yet a significant portion exhibit high-grade tumors associated with poorer outcomes. These findings suggest that while bladder cancer in younger patients tends to be less invasive, aggressive follow-up and treatment are crucial in those with high-grade tumors.

## 1. Introduction

Bladder cancer ranks as the ninth most common malignancy worldwide, contributing significantly to global morbidity and healthcare costs due to its high recurrence and progression rates [1,2,3]. The disease predominantly affects the elderly population, with a median age at diagnosis of approximately 73 years [4,5]. Characterized by a wide spectrum of pathological subtypes and grades, bladder cancer management requires a nuanced approach tailored to tumor behavior and patient factors [6].

In contrast to its prevalence in older adults, bladder cancer in patients under 45 years of age is relatively rare, accounting for only about 1–2% of all cases [7,8]. Young patients often lack the typical risk factors associated with bladder carcinogenesis, such as prolonged tobacco use and occupational exposure to carcinogens like aromatic amines [9,10]. This deviation suggests potential differences in tumor biology and etiology in younger individuals compared to their older counterparts.

The aggressiveness and prognosis of bladder cancer in young patients have been subjects of ongoing debate. Some studies report that younger patients tend to present with less aggressive, low-grade, non-invasive tumors, leading to better clinical outcomes and survival rates [11,12,13]. These findings have been attributed to differences in genetic and molecular tumor profiles, as well as a more robust immune response in younger individuals. Conversely, other research indicates no significant difference in tumor aggressiveness or patient prognosis when compared to older patients, suggesting that age alone may not be a definitive prognostic factor [14,15].

The evidence in the literature highlights a gap in the understanding of the true nature of bladder cancer in young patients. Variability in study designs, sample sizes, and population demographics may contribute to these inconsistencies. Addressing this gap, our study aims to evaluate the clinical and pathological features of bladder cancer in patients under 45 years of age. This population of young age represents a unique demographic, as bladder cancer typically occurs more frequently in older adults. Studying this younger age group can provide valuable insights into the etiological factors and biological behavior distinct from those seen in older cohorts. These younger patients might have different genetic predispositions, environmental exposures, or lifestyle factors contributing to the development of the disease. Understanding these differences could lead to earlier detection strategies, tailored treatment approaches, and potentially better outcomes for this atypical age group affected by bladder cancer. We hypothesize that young patients exhibit distinct tumor characteristics and clinical outcomes compared to the general bladder cancer population. The objective is to analyze a comprehensive cohort of young patients diagnosed with bladder cancer, assessing tumor pathology, treatment modalities, and prognostic outcomes to enhance understanding and inform better management strategies for this demographic.

## 2. Materials and Methods

### 2.1. Study Design and Ethics

This retrospective study included patients who underwent transurethral resection of bladder tumor (TURBT) at the Urology Department of “Pius Brînzeu” County Emergency Clinical Hospital in Timișoara, Romania, between 1 January 2015 and 1 May 2024. Eligible patients were aged 18 to 45 years and had a diagnosis of malignant bladder tumors or papillary urothelial neoplasms of low malignant potential (PUNLMP). The number of patients minimally required to strengthen this study’s validity was calculated to be 60.

This study was conducted in accordance with the Declaration of Helsinki and was approved by the Institutional Review Board of “Pius Brînzeu” County Emergency Clinical Hospital (approval code: 0246; approval date: 15 June 2024). Due to the retrospective nature of this study, informed consent was waived. Patient confidentiality was strictly maintained throughout the research process.

### 2.2. Data Collection and Variables

Data were extracted from hospital medical records and included patient demographics (age and sex), clinical presentation, smoking status, occupational exposure, family history of cancer, tumor characteristics (location, size, histopathology, grade, and stage), treatment modalities, and outcomes (recurrence, progression, and death). Tumor staging was determined using the TNM classification system, and grading followed the World Health Organization (WHO) 2016 criteria, as follows:

T (Tumor): Ta: Non-invasive papillary carcinoma, confined to the bladder lining but not invading the bladder muscle. Tis (Carcinoma in situ): A flat, high-grade tumor that is confined to the bladder lining. T1: The tumor has invaded the connective tissue beneath the bladder lining but not the muscle of the bladder. T2: The tumor has invaded the muscle of the bladder. T2a denotes invasion into the inner half (superficial) of the muscle layer, while T2b denotes invasion into the deep muscle (outer half). T3: The tumor has extended through the muscle layer into the perivesical tissue (tissue surrounding the bladder). T3a indicates microscopic invasion, whereas T3b indicates macroscopic invasion that is visible through imaging or during surgery. T4: The tumor has spread beyond the perivesical tissue to neighboring organs or structures. T4a involves the prostate, uterus, or vagina. T4b involves the pelvic wall or abdominal wall.

N (Node): N0: No regional lymph node involvement. N1: Single regional lymph node involvement in the true pelvis. N2: Multiple regional lymph node involvements in the true pelvis. N3: Involvement of common iliac lymph nodes, beyond the true pelvis. M (Metastasis):

M0: No distant metastasis. M1: Distant metastasis present. M1a refers to distant metastasis confined to the lymph nodes beyond the common iliacs. M1b denotes other distant metastases.

To be included in this study, a patient’s diagnosis had to be confirmed histopathologically following TURBT, and there needed to be complete medical records that detailed patient demographics, tumor characteristics, clinical presentation, treatment modalities, and outcomes. Patients were excluded if they were younger than 18 or older than 45 at the time of diagnosis, were diagnosed with non-malignant bladder conditions, had incomplete medical records, had received bladder cancer treatment before the study period or at other institutions, or had other active malignancies that could confound treatment outcomes and survival analyses. This ensured that this study focused strictly on the specified age group with accurate and complete data, enhancing the reliability of the findings.

### 2.3. Definitions and Outcome Measures

Recurrence was defined as the return of bladder cancer after initial successful treatment, confirmed by histopathological examination. Progression was identified as an increase in tumor stage or grade on subsequent evaluations. Overall survival was calculated from the date of diagnosis to the date of death or last follow-up, while progression-free survival was the time from diagnosis to the occurrence of progression or last follow-up.

### 2.4. Statistical Analysis

The statistical analysis was performed using SPSS software version 25. Continuous variables were expressed as means ± standard deviations or medians with interquartile ranges, depending on data distribution. Categorical variables were presented as frequencies and percentages. Continuous variables were analyzed using the t-test for normally distributed data and the Mann–Whitney U test for non-normally distributed data, facilitating appropriate outlier management and distribution assessment. Categorical variables were evaluated using the chi-square test or Fisher’s exact test, the latter being preferable for small sample sizes to ensure precise evaluation of the independence of categorical factors.

A survival analysis was performed employing the Kaplan–Meier method, and a log-rank test was conducted to assess differences in survival distributions among patient groups, the latter of which is suitable for our dataset including censored observations. To substantiate the validity of our findings and mitigate potential biases, stratification techniques were employed where necessary to control for confounding variables, and sensitivity analyses were conducted to determine the robustness of the results under various assumptions. A *p*-value of less than 0.05 was considered statistically significant.

## 3. Results

### 3.1. Patient Demographics

A total of 60 patients were included, with a mean age of 38.5 ± 5.6 years (range: 22–45 years). There was a male predominance, with 42 males (70%) and 18 females (30%), resulting in a male-to-female ratio of 2.3:1. Smoking was reported in 36 patients (60%), and 10 patients (16.7%) had occupational exposure to bladder carcinogens. A family history of cancer was noted in eight patients (13.3%), as presented in Table 1.

### 3.2. Tumor Characteristics and Treatment Modalities

The majority of tumors were non-muscle-invasive bladder cancer (NMIBC). Specifically, 10 patients (16.7%) had PUNLMP, 30 patients (50%) had stage pTa tumors, and 18 patients (30%) had stage pT1 tumors. Muscle-invasive bladder cancer (MIBC) at stage pT2 was observed in 12 patients (20%). Regarding tumor grade, 34 patients (56.7%) had low-grade tumors, while 26 patients (43.3%) had high-grade tumors. All patients underwent TURBT as the initial treatment. Intravesical therapy with Bacillus Calmette–Guérin (BCG) or chemotherapy agents was administered to 28 patients (46.7%) who had high-grade or recurrent tumors. Radical cystectomy was performed in eight patients (13.3%) with muscle-invasive disease or refractory NMIBC. Adjuvant systemic chemotherapy was provided to six patients (10%) following cystectomy (Table 2).

### 3.3. Clinical Outcomes

Over a median follow-up period of 36 months (range 3–60 months), recurrence occurred in 24 patients (40%), and progression was observed in 10 patients (16.7%). Four patients (6.7%) died due to bladder cancer-related causes. The 3-year overall survival rate was 93.3%, and the 3-year progression-free survival rate was 83.3% (Table 3).

A subgroup analysis demonstrated that patients with high-grade tumors had a significantly higher recurrence rate compared to those with low-grade tumors (61.5% vs. 23.5%, *p* = 0.003). Moreover, patients with muscle-invasive bladder cancer (MIBC) had worse overall survival compared to those with non-muscle-invasive bladder cancer (NMIBC) (75% vs. 100%, *p* = 0.001). Smokers exhibited a higher incidence of high-grade tumors and recurrence, but this difference was not statistically significant (*p* = 0.07), as seen in Table 4.

An analysis of the impact of smoking on clinical outcomes was conducted. Among the 36 smokers, 17 patients (47.2%) experienced tumor recurrence, compared to 7 out of 24 non-smokers (29.2%). Although the smokers had a higher recurrence rate, this difference was not statistically significant (*p* = 0.15). Progression occurred in seven smokers (19.4%) and in three non-smokers (12.5%), which was also not statistically significant (*p* = 0.48).

Clinical outcomes were stratified based on tumor stage at diagnosis. Patients with higher tumor stages exhibited higher rates of recurrence and progression. Specifically, recurrence rates were 10% for PUNLMP, 33.3% for pTa, 55.6% for pT1, and 66.7% for pT2 tumors. Progression was observed in 0% of PUNLMP and pTa patients but increased significantly in pT1 (33.3%) and pT2 (41.7%) patients. The influence of a family history of cancer on clinical outcomes was assessed. Among the eight patients with a positive family history, five (62.5%) experienced recurrence, and three (37.5%) had progression. In comparison, patients without a family history had lower rates of recurrence (38.5%) and progression (15.4%). However, these differences were not statistically significant (recurrence *p* = 0.22; progression *p* = 0.14), as presented in Table 5.

In the youngest cohort (18–29 years), the recurrence and progression rates were the lowest, with only one patient experiencing recurrence and none showing progression. As age increases, there is a marked rise in these rates: the middle age group (30–39 years) shows a recurrence rate of 35.3% and a progression rate of 17.6%, while the oldest group (40–45 years) exhibits even higher rates at 50% and 29.4%, respectively.

Occupational exposure to bladder carcinogens was evaluated in relation to tumor grade. Out of the 10 patients with known occupational exposure, 7 (70%) had high-grade tumors, compared to 19 out of 50 patients (38%) without such exposure. This association was statistically significant (*p* = 0.048), suggesting that occupational exposure may be linked to higher-grade bladder cancers in young patients (Table 6).

Bladder cancer characteristics varied notably across young age groups. The youngest cohort (18–29 years) showed a relatively benign disease profile with only 11.1% recurrence and no progression, suggesting early detection and potentially less aggressive tumors. In contrast, the 30–39 years group experienced increased recurrence (35.3%) and progression rates (17.6%), with 41% having high-grade tumors. The oldest group (40–45 years) faced the most challenging outcomes, with 50% recurrence and 29.4% progression rates, and half the group was diagnosed with high-grade tumors, as presented in Table 7.

The multivariate analysis in Table 8 reveals that age significantly impacted bladder cancer outcomes. Patients aged 30–39 years showed increased recurrence rates (*p* = 0.02) and a mild increase in progression, while those aged 40–45 years experienced significantly higher risks of both recurrence and progression (*p* < 0.001). Additionally, smoking significantly increased recurrence risk (*p* = 0.01), although it did not notably affect progression. Occupational exposure similarly led to increased recurrence (*p* = 0.03) and a mild increase in progression.

### 3.4. Survival Analysis

**Figure 1:**
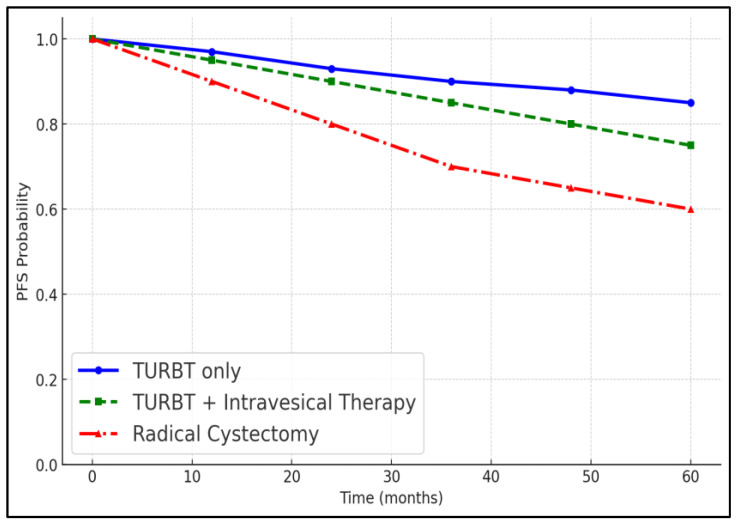
Progression-free survival at 5 years in young patients with bladder cancer.

**Figure 2:**
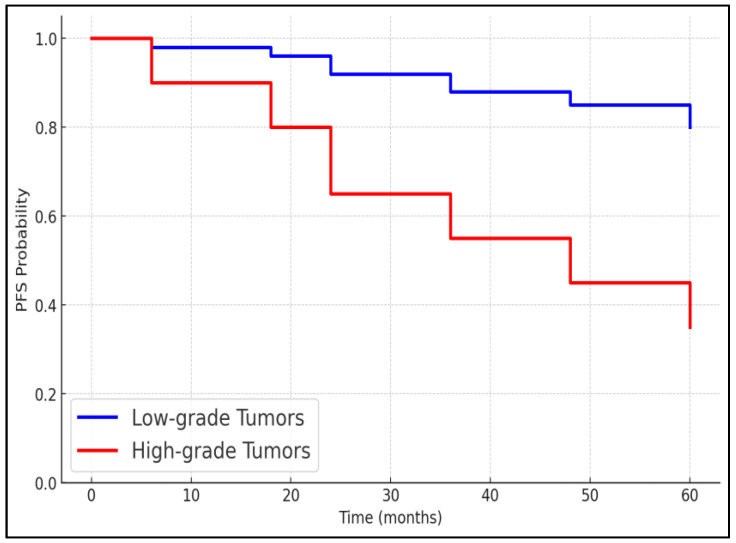
Progression-free survival between low-grade and high-grade tumors in young patients with bladder cancer.

The 3-year overall survival and progression-free survival rates were analyzed based on treatment modalities. Patients treated with TURBT alone had a 3-year overall survival rate of 100% and a progression-free survival rate of 96.9%. Those who received intravesical therapy had slightly lower survival rates, but the differences were not statistically significant. Patients who underwent radical cystectomy had a 3-year overall survival rate of 75%, which was significantly lower compared to those treated with TURBT only (*p* = 0.02), as presented in Table 9, Figure 1 and Figure 2.

## 4. Discussion

The particularity of this study lies in the evaluation of young patients who have been diagnosed with bladder cancer. In the current study, we explored the outcomes of treatment modalities, focusing on the impact of tumor characteristics such as grade and stage, as well as patient demographics including smoking status and occupational exposure. Our results revealed that patients with high-grade tumors showed a recurrence rate significantly higher than those with low-grade tumors (61.5% vs. 23.5%, *p* = 0.003). This finding is consistent with previous studies that suggest that high-grade bladder tumors are more likely to recur and progress, necessitating aggressive management strategies [16]. The distinction between tumor grades in our study underscores the necessity for a tailored approach in the treatment of bladder cancer, where high-grade tumors may benefit from more aggressive initial treatments such as radical cystectomy or systemic chemotherapy.

Furthermore, the survival analysis based on different treatment modalities revealed noteworthy differences. Patients who underwent TURBT alone exhibited excellent survival rates, with a 3-year overall survival of 100% and a progression-free survival of 96.9%. In contrast, those who required radical cystectomy showed significantly lower survival rates (75% overall survival and 62.5% progression-free survival, *p* = 0.028). These findings highlight the effectiveness of TURBT in managing non-muscle-invasive bladder cancer while also pointing to the challenges in treating muscle-invasive disease; however, this difference can also be attributed to the higher cancer stage among those for whom radical cystectomy was necessary [17,18,19]. The lower survival rates associated with radical cystectomy may reflect the advanced nature of disease in these patients and potentially point to the need for improved systemic treatment options post-surgery. Similarly, the 5-year PFS was significantly different in this study among young patients based on treatment modality and tumor grade.

This study also explored the role of smoking and occupational exposure in the prognosis of bladder cancer among young adults. While smokers showed a higher incidence of high-grade tumors and recurrence, these differences were not statistically significant, which might be due to the limited sample size or the younger age profile of the cohort [20,21]. However, occupational exposure was significantly associated with higher-grade tumors (*p* = 0.048), aligning with the existing literature that identifies occupational carcinogen exposure as a critical factor in the etiology of aggressive bladder cancer forms [22,23].

The clinical behavior and prognosis of urothelial bladder cancer in young adults under the age of 40 were explored in two studies, shedding light on the natural history and treatment outcomes of this relatively rare condition in younger patients. In a study conducted by Shahbaz Mehmood et al. [24], a cohort of 55 patients, predominantly male (45 out of 55), were followed for a median of 3.5 years. Their findings revealed that 92.72% of the patients had non-muscle-invasive BC, with notable stability in 65.45% of the patients. However, 26.63% of the patients experienced disease progression, had higher tumor stages and grades, had tumor sizes greater than 3 cm, and were significantly associated with both recurrence (*p* = 0.0431) and progression (*p* = 0.0012). Similarly, a study by Bülent Gunlusoy et al. [25] analyzed 91 patients and found that 91.2% had non-muscle-invasive disease. Despite a lower recurrence rate (18.6%) and progression rate (10.9%) in this larger cohort, no statistically significant differences in recurrence and progression rates between the Ta and T1 stages were noted (*p* = 0.233, *p* = 0.511, respectively).

In the realm of pediatric urology, bladder urothelial neoplasms are notably rare, prompting investigations to better understand their presentation, management, and outcomes. A study by Berrettini et al. [26] focused on a cohort of pediatric patients treated across three tertiary centers from 1999 to 2013, finding that bladder neoplasms, while rare, typically manifested as low-grade and non-muscle-invasive tumors, predominantly as urothelial papillomas (UPs) and papillary urothelial neoplasms of low malignant potential. Their findings suggested a uniform outcome where, after TURBT, none of the patients experienced recurrence or progression during a median follow-up of 5 years, despite varied follow-up protocols across the centers. In a similar manner, a study by Polat et al. [27] reported on 11 pediatric patients diagnosed and treated between 2008 and 2014, also underscoring the rarity and non-aggressive nature of these tumors. Their cohort, all presenting with exophytic tumors treated through TURBT, exhibited no recurrences during follow-up where ultrasonography was primarily utilized.

Fine et al. [28] reported on 23 patients aged 4 to 20 years, where pathological grading revealed a majority of low-grade tumors, including papillary urothelial neoplasms of low malignant potential, with a recurrence rate of only 13%. All patients were alive and disease-free after a median follow-up of 4.5 years, illustrating the benign nature of these tumors when managed appropriately. In a similar manner, Paner et al. [29] conducted a comprehensive review that confirmed the predominance of low-grade and unifocal tumors in patients younger than 40, particularly noting that bladder tumors in the first two decades of life show minimal genetic and epigenetic alterations, contributing to their less aggressive behavior.

In our study, the emergence of high-grade tumors in younger bladder cancer patients contrasts with the expected demographic profiles, where such malignancies are predominantly observed in older cohorts. The implications of this finding suggest a potentially aggressive disease course in younger patients, which necessitates reconsidering current diagnostic and therapeutic strategies. The relevance of MRI-based diagnostics, as discussed in a study by Arita et al. [30], underscores the diagnostic value of the Vesical Imaging-Reporting and Data System (VI-RADS) in differentiating muscle-invasive bladder cancer in variant urothelial carcinomas (VUCs). Our findings resonate with the high diagnostic performance of the VI-RADS, as shown in Arita et al. [30], where the accuracy remains robust across both pure urothelial carcinomas and VUCs, highlighting the potential of the VI-RADS in improving diagnostic accuracy and aiding in the tailored management of this younger subset.

Furthermore, the establishment of the VI-RADS as outlined by Panebianco et al. [31] provides a structured approach to the multiparametric MRI evaluation of bladder cancer, offering detailed insights into tumor characteristics such as stage and muscle invasion potential without radiation exposure. This protocol enhances staging accuracy, which is crucial for determining the appropriate therapeutic approach, particularly in non-muscle-invasive cases where bladder-sparing strategies might be considered. Future research should focus on longitudinal studies to assess the long-term outcomes of MRI-based diagnostic and treatment strategies, particularly for high-grade tumors in younger patients, to optimize care and improve prognoses.

This study on bladder cancer in young patients has several limitations that could impact its broader applicability. The relatively small sample size of 60 patients may not provide enough statistical power to detect significant differences in subgroups or allow for generalizable conclusions. Additionally, this study’s retrospective design introduces potential biases in patient selection and data collection, which could affect outcomes such as treatment efficacy and survival rates. Furthermore, reliance on self-reported data for factors like smoking and family history could lead to inaccuracies. Moreover, additional information regarding sociodemographic factors and smoking behavior was not possible to obtain due to the retrospective nature of this study, therefore, some potential confounding factors might have been missed. Lastly, the absence of data on participants’ socioeconomic status or geographic location omits potentially influential confounding factors. Nevertheless, the rare occurrence of this malignancy in young adults gives an important significance to the findings reported in this study.

## 5. Conclusions

In conclusion, our study emphasizes the critical role of tumor grade in the recurrence and progression of bladder cancer in young patients, highlighting the survival benefits of TURBT treatment in non-muscle-invasive cases. We recommend the implementation of early, precise grading and staging using advanced imaging techniques such as the VI-RADS to optimize treatment outcomes. Additionally, considering the impact of occupational exposure on tumor grade, enhanced preventive measures within occupational health policies are necessary. Future research should focus on larger cohorts and extended follow-ups to refine these tailored management strategies and improve long-term outcomes for this demographic.

## Figures and Tables

**Table 1:** Demographics of young patients undergoing TURBT.

Variable	Value
Total patients	60
Mean age (years)	38.5 ± 5.6
Sex (male/female)	42 (70%)/18 (30%)
Smoking status (smokers)	36 (60%)
Occupational exposure	10 (16.7%)
Family history of cancer	8 (13.3%)

**Table 2:** Tumor characteristics and treatment modalities.

Variables	*n* (%)
Tumor Stage	Number of Patients (%)
PUNLMP	10 (16.7%)
pTa	30 (50%)
pT1	18 (30%)
pT2	12 (20%)
Tumor Grade	Number of Patients (%)
Low-grade	34 (56.7%)
High-grade	26 (43.3%)
Treatment Modality	Number of Patients (%)
TURBT only	32 (53.3%)
TURBT + intravesical therapy	28 (46.7%)
Radical cystectomy	8 (13.3%)
Adjuvant chemotherapy	6 (10%)

TURBT—transurethral resection of bladder tumor; PUNLMP—papillary urothelial neoplasms of low malignant potential.

**Table 3:** Clinical outcomes of young patients undergoing TURBT.

Variables	*n* (%)
Recurrence	24 (40%)
Progression	10 (16.7%)
Disease-specific death	4 (6.7%)
3-Year overall survival	93.30%
3-Year progression-free survival	83.30%

**Table 4:** Subgroup analysis of recurrence based on tumor grade.

Tumor Grade	Number of Patients	Recurrence (%)	*p*-Value
Low-grade	34	8 (23.5%)	
High-grade	26	16 (61.5%)	
*p*-value			0.003

**Table 5:** Recurrence and progression rates.

Variables	Number of Patients	Recurrence (%)	Progression (%)	*p*-Value (Recurrence)	*p*-Value (Progression)
Smoking				0.151	0.483
Smokers	36	17 (47.2%)	7 (19.4%)		
Non-smokers	24	7 (29.2%)	3 (12.5%)		
Tumor Stage				0.044	<0.001
PUNLMP	10	1 (10%)	0 (0%)		
pTa	30	10 (33.3%)	0 (0%)		
pT1	18	10 (55.6%)	6 (33.3%)		
pT2	12	8 (66.7%)	5 (41.7%)		
Total	60	29 (48.3%)	11 (18.3%)		
Family History				0.229	0.146
Positive	8	5 (62.5%)	3 (37.5%)		
Negative	52	20 (38.5%)	8 (15.4%)		
Age Groups					
18–29	9	11.1% (1)	0% (0)		
30–39	17	35.3% (6)	17.6% (3)	0.042	0.068
40–45	34	50% (17)	29.4% (10)	0.015	0.021

**Table 6:** Tumor grade based on occupational exposure.

Occupational Exposure	Number of Patients	High-Grade Tumors (%)	Low-Grade Tumors (%)	*p*-Value
*p*-value				0.048
Yes	10	7 (70%)	3 (30%)	
No	50	19 (38%)	31 (62%)	

**Table 7:** Bladder cancer characteristics by age group.

Age Group (Years)	Number of Patients	Tumor Grade (High/Low)	Tumor Stage (PUNLMP, pTa, pT1, pT2)	Family History (Yes/No)	Recurrence Rate (%)	Progression Rate (%)
18–29	9	3 High/6 Low	1 PUNLMP, 3 pTa, 3 pT1, 2 pT2	1 Yes/8 No	11.10%	0%
30–39	17	7 High/10 Low	3 PUNLMP, 7 pTa, 4 pT1, 3 pT2	3 Yes/14 No	35.30%	17.60%
40–45	34	16 High/18 Low	6 PUNLMP, 20 pTa, 11 pT1, 7 pT2	4 Yes/30 No	50%	29.40%

**Table 8:** Multivariate analysis of factors affecting recurrence and progression in bladder cancer patients.

Variable	Coefficient	Standard Error	*p*-Value	95% Confidence Interval	Impact on Recurrence	Impact on Progression
Age						
18–29 years	Ref.					
30–39 years	0.833	0.21	0.02	0.46 to 1.29	Increased	Mildly increased
40–45 years	1.417	0.35	<0.001	0.86 to 2.03	Significantly increased	Significantly increased
Smoking Status						
Non-smoker	Ref.					
Smoker	0.522	0.10	0.01	0.33 to 0.75	Increased	No significant impact
Occupational Exposure						
No exposure	Ref.					
Exposure	0.784	0.26	0.03	0.93 to 1.16	Increased	Mildly increased

**Table 9:** Survival rates based on treatment modality.

Treatment Modality	Number of Patients	3-Year OS (%)	3-Year PFS (%)	*p*-Value (OS)
TURBT only	32	100%	96.9%	
TURBT + intravesical therapy	28	96.4%	89.3%	0.311
Radical cystectomy	8	75%	62.5%	0.028

TURBT—transurethral resection of bladder tumor; OS—overall survival.

## Data Availability

The data presented in this study are available on request from the corresponding author. The data are not publicly available due to patient privacy standards that regulate the handling of clinical data.

## References

[1] Halaseh S.A., Halaseh S., Alali Y., Ashour M.E., Alharayzah M.J. (2022). A Review of the Etiology and Epidemiology of Bladder Cancer: All You Need To Know. Cureus.

[2] Richters A., Aben K.K.H., Kiemeney L.A.L.M. (2020). The global burden of urinary bladder cancer: An update. World J. Urol..

[3] Zhang Y., Rumgay H., Li M., Yu H., Pan H., Ni J. (2023). The global landscape of bladder cancer incidence and mortality in 2020 and projections to 2040. J. Glob. Health.

[4] Ferlay J., Soerjomataram I., Dikshit R., Eser S., Mathers C., Rebelo M., Parkin D.M., Forman D., Bray F. (2015). Cancer incidence and mortality worldwide: Sources, methods and major patterns in GLOBOCAN 2012. Int. J. Cancer.

[5] Siegel R.L., Miller K.D., Jemal A. (2019). Cancer statistics, 2019. CA Cancer J. Clin..

[6] Guo C.C., Shen S.S., Czerniak B. (2023). Recent Advances in the Classification of Bladder Cancer—Updates from the 5th Edition of the World Health Organization Classification of the Urinary and Male Genital Tumors. Bladder Cancer.

[7] Albakri M., Abu-Hijlih R., Salah S., Al-Ibraheem A., Abuhijla F., Serhan H.A., Farkouh A., Obeid Z., Shahait M. (2023). Bladder cancer in young adults: Disease and treatment characteristics of patients treated at a tertiary cancer center. Urol. Ann..

[8] Guo C.C., Czerniak B. (2023). Updates of Prostate Cancer from the 2022 World Health Organization Classification of the Urinary and Male Genital Tumors. J. Clin. Transl. Pathol..

[9] Freedman N.D., Silverman D.T., Hollenbeck A.R., Schatzkin A., Abnet C.C. (2011). Association between smoking and risk of bladder cancer among men and women. JAMA.

[10] Inoue-Choi M., Hartge P., Liao L.M., Caporaso N., Freedman N.D. (2018). Association between long-term low-intensity cigarette smoking and incidence of smoking-related cancer in the national institutes of health-AARP cohort. Int. J. Cancer.

[11] Erlich A., Zlotta A.R. (2016). Treatment of bladder cancer in the elderly. Investig. Clin. Urol..

[12] Zhong H., George S., Kauffman E., Guru K., Azabdaftari G., Xu B. (2014). Clinicopathologic characterization of intradiverticular carcinoma of urinary bladder—A study of 22 cases from a single cancer center. Diagn. Pathol..

[13] Chang W.C., Chang Y.H., Pan C.C. (2012). Prognostic significance in substaging ofT1 urinary bladder urothelial carcinoma on transurethral resection. Am. J. Surg. Pathol..

[14] Noon A.P., Albertsen P.C., Thomas F., Rosario D.J., Catto J.W. (2013). Competing mortality in patients diagnosed with bladder cancer: Evidence of undertreatment in the elderly and female patients. Br. J. Cancer.

[15] Moschini M., Martini A., Zamboni S., Mattei A., Baumeister P., Di Bona C., Dell’Oglio P., Zaffuto E., Burgio G., Shariat S.F. (2019). Evaluation of Cause of Death After Radical Cystectomy for Patients With Bladder Cancer: The Impact of Age at the Time of Surgery. Clin. Genitourin. Cancer.

[16] Azhar R.A., Nassir A.M., Saada H., Munshi S., Alghamdi M.M., Bugis A.M., Elkoushy M.A. (2021). High-Grade Non-Muscle Invasive Bladder Cancer: When to Move to Early Radical Cystectomy?. Cureus.

[17] Ping Z., Zhan X., Chen T., Zheng Y., Jiang M., Li Y., Fu B. (2022). Survival Outcome of Partial Cystectomy versus Transurethral Bladder Tumor Resection in T1 High-Grade Bladder Cancer Patients: A Propensity Score Matching Study. J. Oncol..

[18] Kim L.H.C., Patel M.I. (2020). Transurethral resection of bladder tumour (TURBT). Transl. Androl. Urol..

[19] Grabe-Heyne K., Henne C., Mariappan P., Geiges G., Pöhlmann J., Pollock R.F. (2023). Intermediate and high-risk non-muscle-invasive bladder cancer: An overview of epidemiology, burden, and unmet needs. Front. Oncol..

[20] Kumar R., Matulewicz R., Mari A., Moschini M., Ghodoussipour S., Pradere B., Rink M., Autorino R., Desai M.M., Gill I. (2023). Impact of smoking on urologic cancers: A snapshot of current evidence. World J. Urol..

[21] Jiang X., Castelao J.E., Yuan J.M., Stern M.C., Conti D.V., Cortessis V.K., Pike M.C., Gago-Dominguez M. (2012). Cigarette smoking and subtypes of bladder cancer. Int. J. Cancer.

[22] Xie S., Friesen M.C., Baris D., Schwenn M., Rothman N., Johnson A., Karagas M.R., Silverman D.T., Koutros S. (2024). Occupational exposure to organic solvents and risk of bladder cancer. J. Expo. Sci. Environ. Epidemiol..

[23] Shala N.K., Stenehjem J.S., Babigumira R., Liu F.C., Berge L.A.M., Silverman D.T., Friesen M.C., Rothman N., Lan Q., Hosgood H.D. (2023). Exposure to benzene and other hydrocarbons and risk of bladder cancer among male offshore petroleum workers. Br. J. Cancer.

[24] Mehmood S., Alothman K.I., Al Rumayyan M., Altaweel W.M., Alhussain T.O. (2022). Clinical behavior and survival outcome of urothelial bladder cancer in young adults. Urol. Ann..

[25] Gunlusoy B., Ceylan Y., Degirmenci T., Kozacioglu Z., Yonguc T., Bozkurt H., Aydogdu O., Sen V. (2015). Urothelial bladder cancer in young adults: Diagnosis, treatment and clinical behaviour. Can. Urol. Assoc. J..

[26] Berrettini A., Castagnetti M., Salerno A., Nappo S.G., Manzoni G., Rigamonti W., Caione P. (2015). Bladder urothelial neoplasms in pediatric age: Experience at three tertiary centers. J. Pediatr. Urol..

[27] Polat H., Utangac M.M., Gulpinar M.T., Cift A., Erdogdu I.H., Turkcu G. (2016). Urothelial neoplasm of the bladder in childhood and adolescence: A rare disease. Int. Braz. J. Urol..

[28] Fine S.W., Humphrey P.A., Dehner L.P., Amin M.B., Epstein J.I. (2005). Urothelial neoplasms in patients 20 years or younger: A clinicopathological analysis using the world health organization 2004 bladder consensus classification. J. Urol..

[29] Paner G.P., Zehnder P., Amin A.M., Husain A.N., Desai M.M. (2011). Urothelial neoplasms of the urinary bladder occurring in young adult and pediatric patients: A comprehensive review of literature with implications for patient management. Adv. Anat. Pathol..

[30] Arita Y., Yoshida S., Shigeta K., Kwee T.C., Edo H., Okawara N., Hashimoto M., Ishii R., Ueda R., Mikami S. (2023). Diagnostic Value of the Vesical Imaging-Reporting and Data System in Bladder Urothelial Carcinoma with Variant Histology. Eur. Urol. Oncol..

[31] Panebianco V., Narumi Y., Altun E., Bochner B.H., Efstathiou J.A., Hafeez S., Huddart R., Kennish S., Lerner S., Montironi R. (2018). Multiparametric Magnetic Resonance Imaging for Bladder Cancer: Development of VI-RADS (Vesical Imaging-Reporting And Data System). Eur. Urol..

